# Micronodular thymoma with lymphoid stroma: Contrast-enhanced CT features with histopathological correlation in 10 patients

**DOI:** 10.3389/fonc.2022.964882

**Published:** 2022-08-30

**Authors:** Lei Miao, Lin Yang, Jia-Xing Zhang, Xu-Jie Sun, Huan-Huan Zhang, Lin-Lin Qi, Meng Li

**Affiliations:** ^1^Departments of Radiology, National Cancer Center/National Clinical Research Center for Cancer/Cancer Hospital, Chinese Academy of Medical Sciences and Peking Union Medical College, Beijing, China; ^2^Departments of Pathology, National Cancer Center/National Clinical Research Center for Cancer/Cancer Hospital, Chinese Academy of Medical Sciences and Peking Union Medical College, Beijing, China

**Keywords:** CT, thymic tumor, pathology, mediastinum, diagnosis

## Abstract

**Objectives:**

This study aimed to evaluate and summarize the contrast-enhanced computed tomography (CECT) imaging features of micronodular thymoma with lymphoid stroma (MTWLS) based on all MTWLS patients at our institution and was the first imaging study of MTWLS worldwide.

**Methods:**

This retrospective study included 10 MTWLS patients who underwent CECT between April 2012 and November 2021. We collected and analyzed the CECT imaging features, including the location, size, shape, tumor density, classification, and CT value of the solid component. Descriptive statistical analysis was performed using the SPSS software (version 26.0; IBM).

**Results:**

Ten patients (five males [50%], five females [50%]; median age, 61.4 years; range, 54-72 years) underwent CECT. Of the 10 cases, one case was purely cystic, seven cases were cystic-solid, and two cases were purely solid. Six cases were round/oval in shape, and four cases were irregularly shaped. Excluding a purely cystic tumor with an unmeasurable degree of enhancement, two cases showed moderate enhancement, and seven cases showed significant enhancement. Among the solid or cystic-solid cases, the mean CT value of the measurable solid component on the enhanced scan was 93.9 HU. Nine masses were located adjacent to the mediastinal pleura, pericardium, or large vessels. Additionally, there were no malignant tumor signs in any patient, including penetration of the mediastinal pleura or involvement of the pericardium, pleural effusion, elevation of the diaphragm, or direct vascular invasion.

**Conclusion:**

MTWLS demonstrates certain features on CECT, such as a high rate of cystic change, significant solid component enhancement, and no malignant, invasive imaging features. These CECT features are helpful for diagnosing MTWLS.

## Introduction

Micronodular thymoma with lymphoid stroma (MTWLS) is a rare subtype of thymoma and accounts for 1-5% of cases (1). According to the WHO (5th edition in 2021) classification, its ICD-O value is 1, which means unspecified, borderline, or uncertain behavior (2). MTWLS was discovered in 1999 by Suster and Moran (3); at that time, it was defined as micronodular thymoma with lymphoid B-cell hyperplasia, but today, this name is no longer recommended. Some studies also use the abbreviations “MNT” or “MNTLS” to represent MTWLS (1, 4). MTWLS is in a phase of constantly being known and recognized.

Previous studies on MTWLS were mostly limited to the description of pathology and certain clinical features, but no imaging studies have been conducted. Contrast-enhanced computed tomography (CECT) is the most commonly used examination method for mediastinal space-occupying lesions. It can indicate the location, morphological features, and enhanced density of the tumor and can assist in the differential diagnosis through some imaging signs (5). This study aims to discuss the CECT imaging features of MTWLS, analyze its relationship with pathological features to improve the preoperative diagnosis rate of the disease by CECT imaging, provide an important reference for the timing of clinical treatment and the selection of treatment plans, and improve doctors’ clinical and imaging understanding of the disease.

## Methods

### Patient

**Figure 1:**
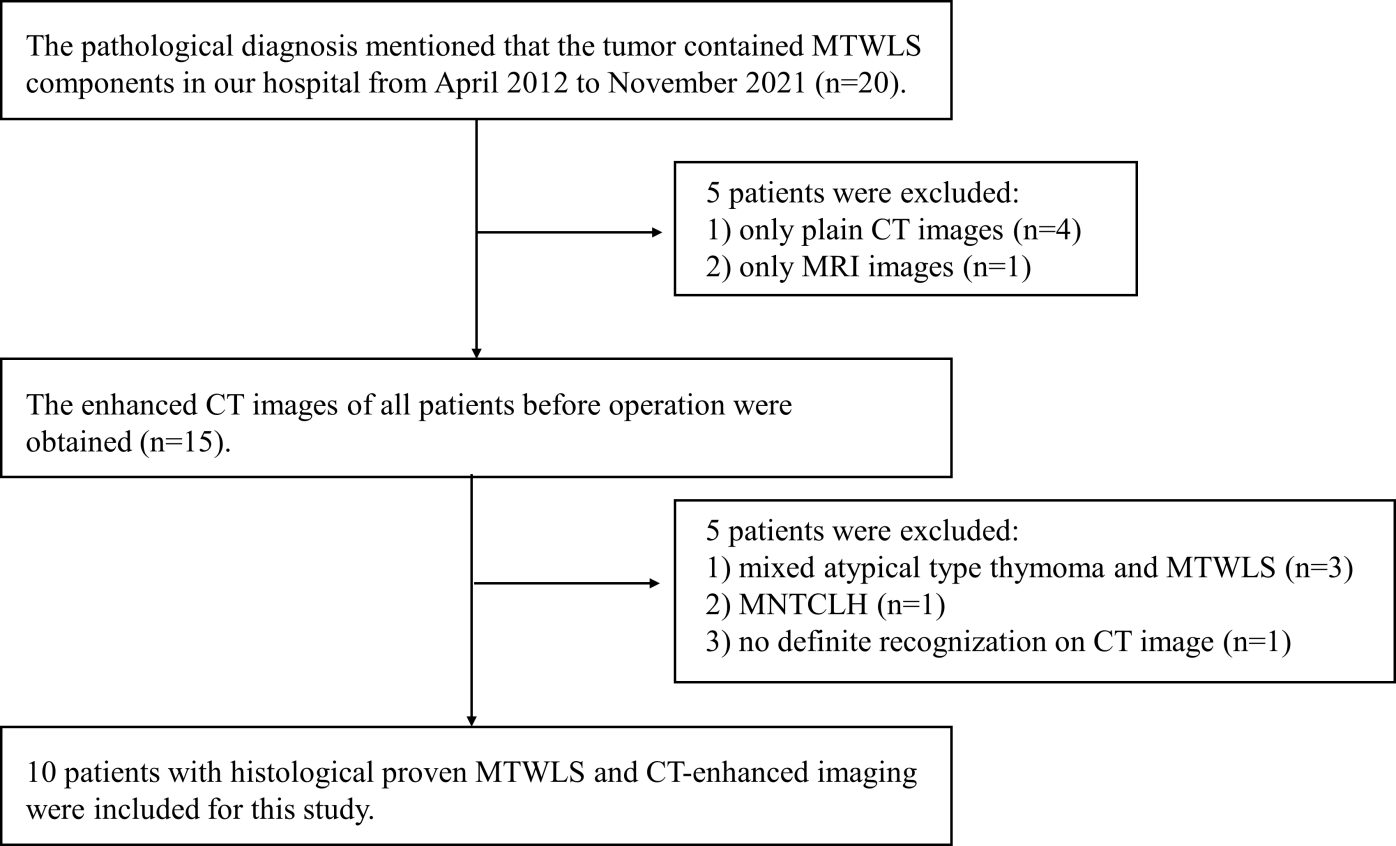
Flow diagram of the study cohort.

This retrospective study was approved by the local institutional review board, and written informed consent was waived. The clinical, imaging and pathological data of MTWLS patients diagnosed and treated in our hospital from April 2012 to November 2021 were retrospectively analyzed. The inclusion criteria were as follows: (1) complete surgical resection and definitive pathological diagnosis; and (2) CECT examination at our hospital before surgery. The exclusion criteria were as follows: (1) incomplete imaging data; (2) pathological diagnosis of mixed atypical thymoma and MTWLS or micronodular thymic carcinoma with lymphoid hyperplasia (MNTCLH); and (3) occasional microscopic MTWLS after thymic cyst surgery, with no definite recognition on CECT. The flow diagram of the study cohort is shown in **Figure 1**.

### Clinical data and pathological evaluation

All pathological data were reviewed by experienced pathologists in the thoracic oncology subspecialty. After surgical resection, the specimens were fixed in neutral buffered formalin, routinely dehydrated, and embedded in paraffin. Then, 4-µm-thick sections were obtained, stained with HE and observed under a microscope. The recorded clinical data included the patient’s age, sex, tumor discovery process, symptoms, presence of myasthenia gravis, hypertension, smoking, drinking, family history, CA199, AFP, CEA, β-HCG, TNM stage, and operation time. The TNM stage was determined according to the American Joint Committee on Cancer (AJCC) Staging Manual, 8^th^ edition. Follow-up and recurrence information were obtained from physician or telephone interviews; the last follow-up was in March 2022. Pathomorphological records included whether the mass involved the capsule, surrounding fat, or mediastinal pleura and whether there was a cystic component and Masaoka stage. Immunohistochemistry was performed for CD117, CK19, CK7, EMA, Ki-67, P63, and CD5 as epithelial markers and CD1a, TDT, CD3, CD5, and CD20 as lymphocytic markers.

### CT scan acquisition

All patients underwent CECT examination. CT images were acquired with multidetector CT scanners (Revolution EVO CT, Optima CT 660, Discovery CT 750, GE Healthcare Milwaukee, WI, USA, or IQon Spectral CT, Philips Medical Systems, Cleveland, OH, USA). Scanning parameters: tube voltage 120 kVp, tube current 200~350 mAs, scanning slice thickness 5 mm, reconstruction interval 5 mm. A standard reconstruction algorithm was used, including a slice thickness of 1.25 mm or 1 mm according to the needs of multiplane reconstruction. For the enhanced chest CT scan, a bolus of the contrast agent iomeprol was intravenously injected at an iodine concentration of 300 mg/ml, a dose of 85 ml, a flow rate of 2.5 ml/s, and a delay of 35 s prior to imaging. A mediastinal window (width 350 HU, level 40 HU) was used to observe and analyze images.

### Image analysis

All images were passed to the picture archiving and communication system (PACS) and ADW4.7 workstation (GE Healthcare, Milwaukee, WI, USA). Two radiologists (with 2 years and 8 years of experience in chest imaging diagnosis) read the images of the enrolled patients repeatedly under the mediastinal window together, and the results were finally determined by a chief radiologist with 20 years of experience in chest imaging diagnosis. The normative and conventional imaging findings of the recorded imaging data were described according to the guidelines of the International Thymic Malignancy Imaging Group (ITMIG), including the location of the tumor on the anterior mediastinum (left, middle, right); size of the tumor (long diameter, short diameter, longitudinal diameter); tumor shape (round or oval/irregular); tumor density classification (cystic/solid/cystic-solid); CT value of the solid component; presence of multiple cystic areas; CT value of the back muscle at the same level; whether the tumor is located adjacent to or invades the mediastinal pleura, pericardium or big vessels; presence of pleural effusion, elevation of the diaphragm, and mediastinal lymph node enlargement.

The tumor was defined as having a cystic component if it had a CT value of 0-20 HU and a clearly demarcated and measurable area. The area with the most significant tumor enhancement was taken as the CT value of the solid component. The enhancement degree of the solid component of the tumor was divided into mild (enhancement degree less than that of the back muscles at the same level by at least 20 HU), moderate (enhancement degree within 20 HU of that of the back muscles at the same level) and significant (enhancement degree more than that of the back muscles by at least 20 HU). Measurements of all quantitative characteristics were separately measured by two radiologists, and their averages were calculated to ensure the reliability of the measurements. We observed the relationship between the mass and the mediastinum, pericardium or large vessels on coronal, sagittal and axial views. If there was no fat space between the mass and the mediastinal pleura, pericardium or large vessels, we defined the mass as being located adjacent to these structures. If the tumor directly extended into the mediastinal pleura, pericardium or large vessels, it was defined as invading these structures.

### Statistical analysis

Data were analyzed by using SPSS software (version 26.0; IBM). Continuous variables are expressed as medians and ranges. Categorical variables are expressed as frequencies and proportions.

## Results

### Clinical characteristics

In total, 10 patients (5 male patients [50%]; median age, 61.4 years; age range 54-72 years) were included. Nine patients were found during incidental finding on CT, and there were no obvious clinical symptoms (chest pain, chest tightness, cough and anterior chest discomfort, etc.). One patient (case 4) was incidentally found during a preoperative CT examination for lung cancer, and the symptoms of hemoptysis were caused by the primary lung cancer. No symptoms of myasthenia gravis were observed in any patients. Six patients had hypertension, 5 patients had a smoking history, and 5 patients had a drinking history. None of the blood tumor markers detected by the laboratory in this study were positive. The TNM staging indicated that all cases were T1a. **Table 1** briefly lists the clinical and pathological features of the 10 patients.

**Table 1:** Clinical and Pathological Features of MTWLS.

Case	Sex	Age	Symptom	Myasthenia Gravis	Operation	Operation Date	Capsular Invasion	Cystic Component	Surrounding Fat Invasion	TNM-Stage	Masaoka-Stage
1	F	55	-	-	VATS	9/7/2021	-	+	NA	T1a	1
2	M	57	-	-	VATS	11/26/2021	-	+	-	T1a	1
3	M	63	-	-	Thoracotomy	11/30/2021	-	+	NA	T1a	1
4	M	56	Hemoptysis	-	VATS	1/4/2018	+	+	+	T1a	2
5	F	55	-	-	VATS	1/22/2018	+	-	-	T1a	2
6	M	54	-	-	VATS	3/19/2018	+	+	+	T1a	2
7	M	65	-	-	VATS	12/31/2015	-	+	-	T1a	1
8	F	71	-	-	VATS	3/16/2015	+	+	+	T1a	2
9	F	66	-	-	VATS	9/1/2013	+	+	+	T1a	2
10	F	72	-	-	VATS	3/21/2019	-	+	NA	T1a	1

— NA, Not available.

### Pathological features

A microscopic view of the tissue specimens shows that the tumor had a clear boundary under the microscope, some visible fibrous capsules, epithelioid tumor cells distributed in a micronodular shape in the tumor area, aggregation of B lymphocytes in some areas, and abundant lymphocytic interstitial segmentation within the interstitium. Among the cases, three had intact tumor capsules, five had tumors involving the capsule, four involved the surrounding fat, and nine had cystic components. Tissue microscopy showed no tumors involving the mediastinal pleura. Masaoka pathological staging indicated that five cases were in stage I, and five cases were in stage II. Seven patients underwent immunohistochemistry. The positive rate of CD20 in the interstitium was 100% (4/4), and other immunohistochemical indicators and microscopic observations are shown in the **Supplementary Materials**.

### Imaging findings

**Figure 2:**
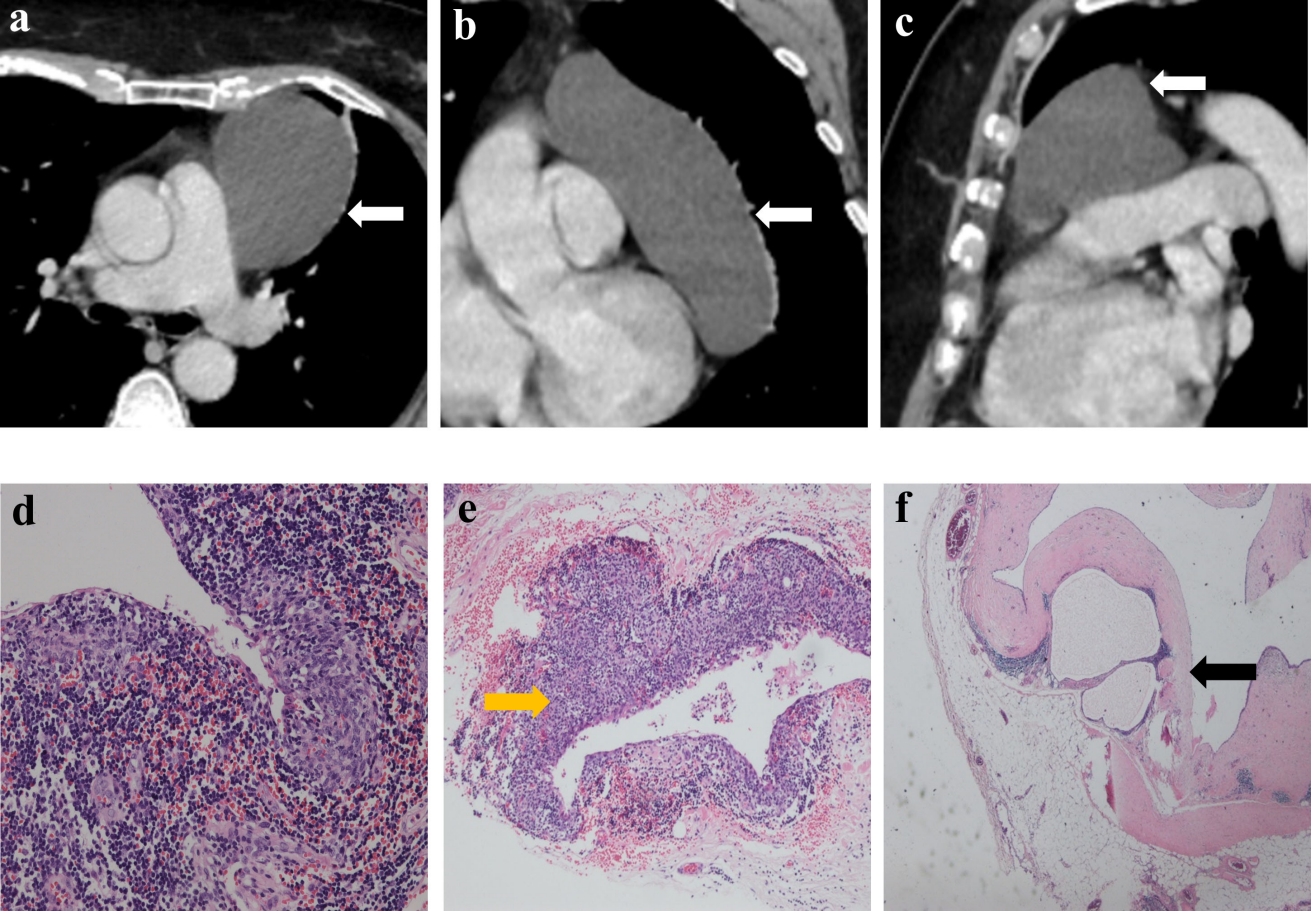
Imaging and histologic findings of a 55-year-old woman with primary mediastinal MTWLS (case 1). **(A)** Axial, **(B)** coronal and **(C)** sagittal chest CT images with contrast enhancement reveal a 7.6×5.9×12.2-cm purely cystic and round or oval left anterior mediastinal mass (white arrow) adjacent to the mediastinum pleura, pericardium and large blood vessels. **(D)** Hematoxylin-eosin (H-E)–stained section of the mass reveals spindle cells forming multiple micronodules separated by abundant interstitial lymphocytes. **(E)** Some cyst walls (yellow arrow) were covered with nodular tumors **(F)** and other cyst walls were covered with a single layer of epithelioid cells (black arrow).

**Figure 3:**
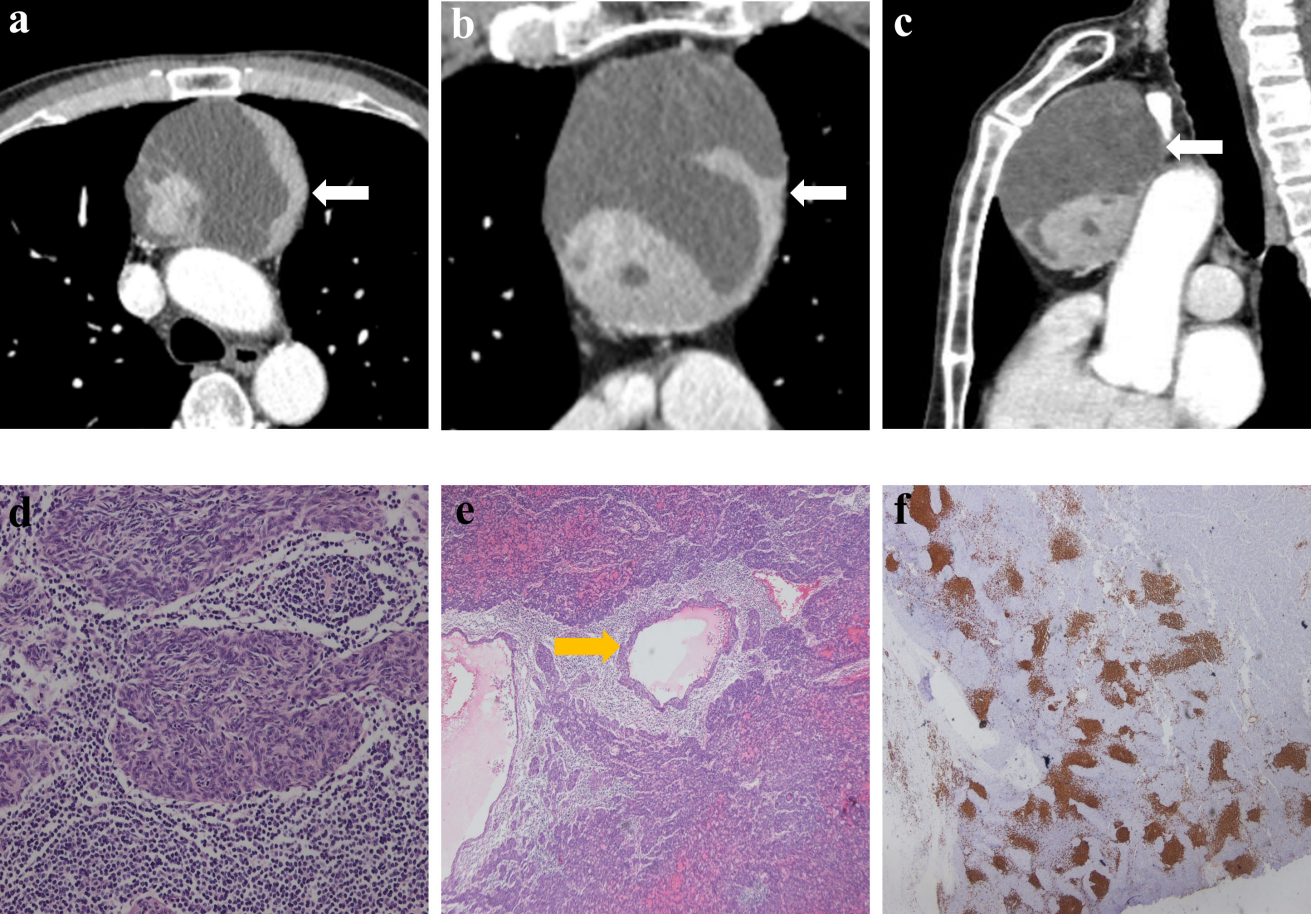
Imaging and histologic findings in a 63-year-old man with primary mediastinal MTWLS (case 3). **(A)** Axial, **(B)** coronal and **(C)** sagittal chest CT images with contrast enhancement reveal a 7.3×7.0×9.0-cm multiple cystic region and a round or oval middle anterior mediastinal mass (white arrow) adjacent to the mediastinum pleura and large blood vessels. The measurable solid components showed significant enhancement. **(D)** Hematoxylin-eosin (H-E)–stained section of the mass reveals spindle cells forming multiple micronodules separated by abundant interstitial lymphocytes. **(E)** Cyst walls (yellow arrow) were covered with nodular tumor. **(F)** Lymphocytes had diffusely strong positive immunostaining for CD20 (= B cells), and the epithelial cell nests were negative for CD20.

**Figure 4:**
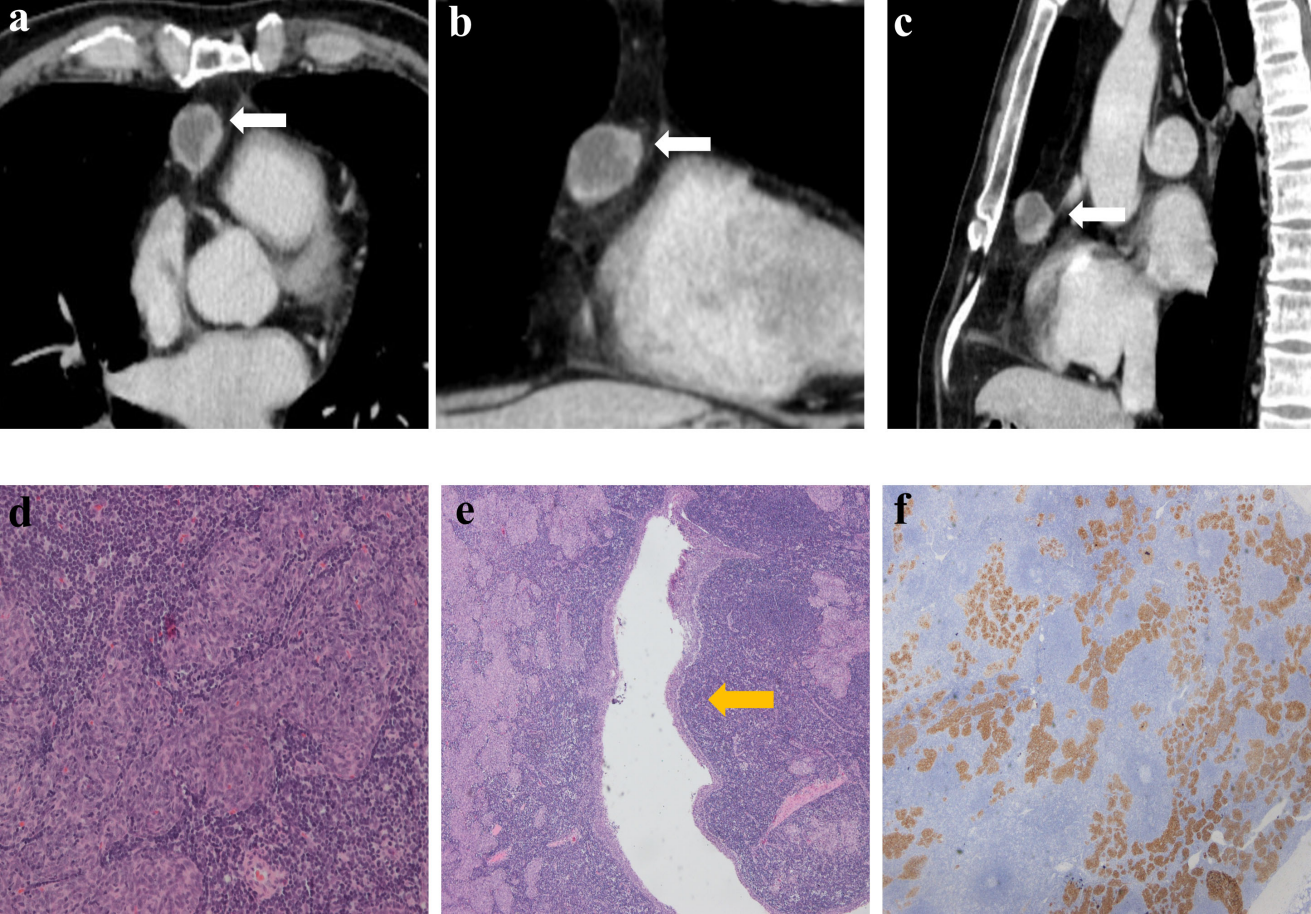
Imaging and histologic findings in a 56-year-old man with primary mediastinal MTWLS (case 4). **(A)** Axial, **(B)** coronal and **(C)** sagittal chest CT images with contrast enhancement reveal a 2.2×2.1×2.6 cm multiple cystic region and round or oval middle anterior mediastinal mass (white arrow) adjacent to the mediastinum pleura. It had similar imaging findings to the annular enhancement. **(D)** Hematoxylin-eosin (H-E)–stained section of the mass reveals spindle cells forming multiple micronodules separated by abundant interstitial lymphocytes. **(E)** Cyst walls (yellow arrow) were covered with nodular tumor. **(F)** The epithelial component was positive for CK7.

**Table 2** lists the CECT imaging features of all patients. For all 10 patients, the cases were located in the anterior mediastinum, four (40%) on the right, two (20%) in the middle, and four (20%) on the left. Six patients had tumors with round/oval shapes (60%), and four had irregularly shaped tumors (40%). The average long diameter of the 10 cases as measured on CECT was 3.9 cm. One case (**Figure 2**) was purely cystic (10%), seven were cystic-solid (70%), and two were purely solid (20%). Six of the seven cystic-solid cases (**Figure 3**) showed multiple isolated cystic areas (85.7%). Two patients showed moderate enhancement (20%), seven showed significant enhancement (70%), and one patient showed purely cysitc (10%) (**Figure 2**). The mean CT value of the measurable solid components of the solid or cystic-solid cases on the enhanced scans was 93.9 HU. Four patients (cases 2, 4, 11, and 12) had similar imaging findings to the annular enhancement (**Figure 4**). The tumor was located adjacent to the mediastinal pleura, pericardium or large vessels in nine patients (90%). No tumor penetrated the mediastinal pleura or involved the pericardium for any patient, and no tumor showed signs of malignancy, such as pleural effusion, elevation of the diaphragm, direct invasion of the vascular cavity, or mediastinal lymph node enlargement.

**Table 2:** CECT Imaging Features of MTWLS.

Case	Location	Long Diameter (cm)	Short Diameter (cm)	Vertical Diameter (cm)	Cystic/Cystic-Solid/Solid	Solid Component CT Value	CT Value of Back Muscle in Same Slice	Multiple Cystic Region	Adjacent to Mediastinum, Pleura, Pericardium or Big Blood Vessels	Mediastinum, Pleura, Pericardium or Big Blood Vessel Invasion	Shape
1	Left	7.6	5.9	12.2	C	NA	NA	–	+	–	R
2	Left	1.6	1.2	1.3	CS	67	68	+	–	–	I
3	Middle	7.3	7.0	9.0	CS	107	63	+	+	–	R
4	Right	2.2	2.1	2.6	CS	91	66	–	+	–	R
5	Left	2.7	2.2	3.7	S	133	80	–	+	–	R
6	Middle	1.4	1.3	1.6	S	104	71	–	+	–	R
7	Right	2.8	1.8	2.5	CS	108	68	+	+	–	I
8	Right	5.5	4.7	6.5	CS	88	50	+	+	–	I
9	Right	4.1	3	4.8	CS	96	68	+	+	–	R
10	Left	3.9	2.4	5.4	CS	51	43	+	+	–	I

— C, cystic; CS, cystic-solid; I, irregular; R, round or oval; S, solid; NA, Not available.

### Comparative study of the CECT findings and pathology of MTWLS

**Figure 5:**
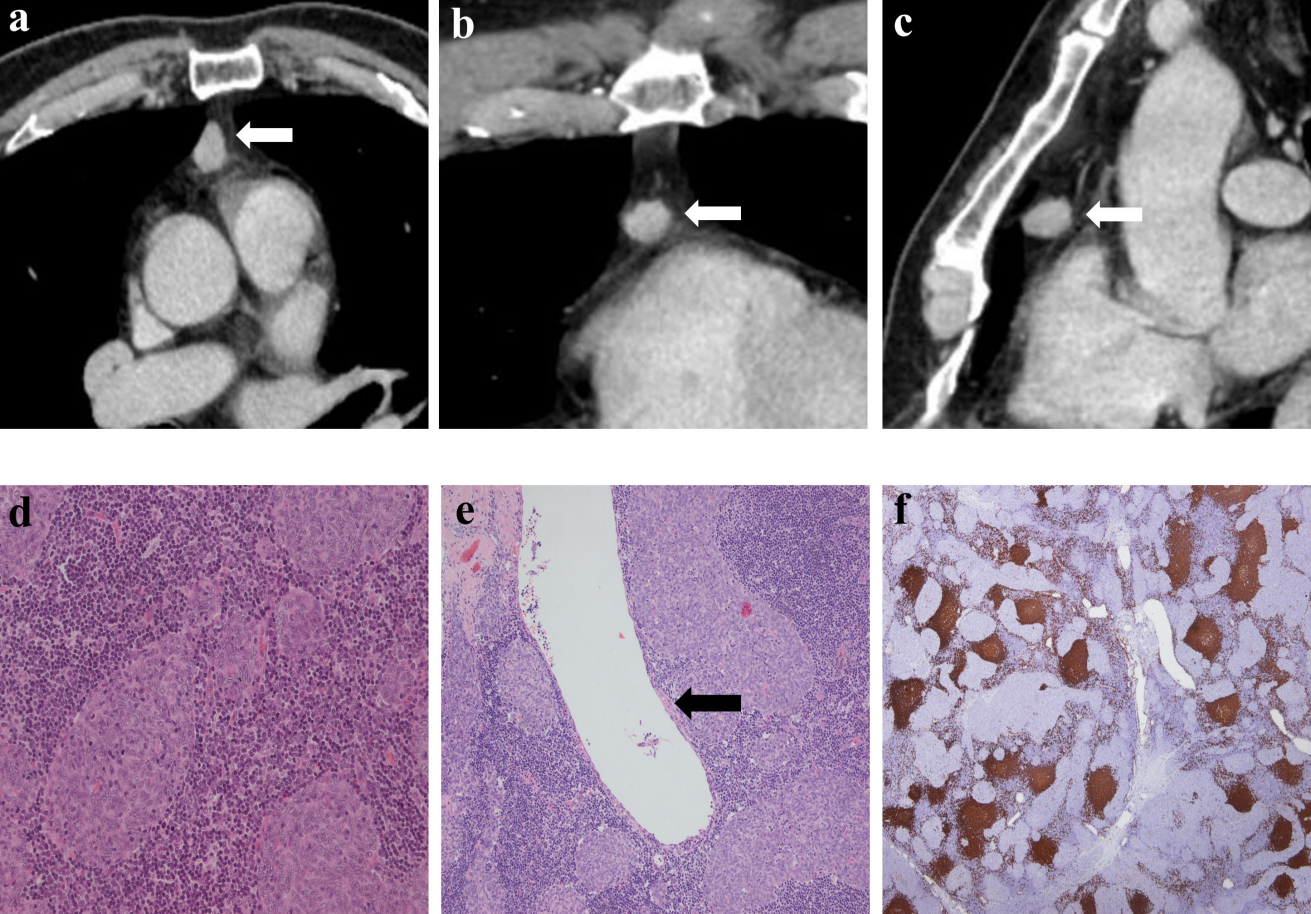
Imaging and histologic findings in a 54-year-old man with primary mediastinal MTWLS (case 6). **(A)** Axial, **(B)** coronal and **(C)** sagittal chest CT images with contrast enhancement reveal a 1.4×1.3×1.6 cm solid and round or oval middle anterior mediastinal mass (white arrow) adjacent to the mediastinum pleura. The measurable solid components showed significant enhancement. **(D)** Hematoxylin-eosin (H-E)–stained section of the mass reveals spindle cells forming multiple micronodules separated by abundant interstitial lymphocytes. **(E)** Some cystic regions not found by CT were visible under the microscope, and cyst walls (black arrow) were covered with a single layer of epithelioid cells. **(F)** Lymphocytes had diffusely strong positive immunostaining for CD20 (= B cells), and the epithelial cell nests were negative for CD20.

The solid components of all patients on CECT corresponded to the micronodular distribution of tumor cells and abundant lymphocytic stroma. The cystic components that appeared on CECT in all patients corresponded to purely cystic fluid instead of liquefied necrosis on pathology. Some cyst walls (cases 1, 2, 6, 7, and 10) were covered with a single layer of or pseudostratified epithelioid cells, and others (cases 1, 2, 3, 4, 7, and 8) were covered with nodular tumor cells. Only one patient (case 6) (**Figure 5**) showed a purely solid tumor on CECT, but microscopically, a cystic component was visible.

### Treatment and follow-up data

All patients underwent surgery in our hospital, including video-assisted thoracic surgery (VATS) (n=9) and thoracotomy (n=1), and no serious complications occurred in any of the patients. The final postoperative TNM tumor stage for all patients in this study was T_1_N_0_M_0._ The patients were followed up after surgery, and no recurrence was found in any patients until the end of the follow-up period.

## Discussion

This study reviewed and summarized the CECT imaging features and clinical and pathological data of 10 patients with MTWLS and is the first systematic imaging study of this disease. Three main principal findings can be derived. First, MTWLS are mostly cystic-solid, and some have multiple cystic components. Second, the solid component of MTWLS is significantly enhanced. Finally, MTWLS has no obvious malignant or invasive CECT imaging features. These new imaging findings and insights can help diagnose MTWLS.

In our study, the most important CECT feature of MTWLS was the high rate of cystic changes (8/10; 80%). The cystic components found on CECT in our study clearly correspond with the pathological findings observed by microscopy; additionally, in one patient, the cystic components were found on pathology but were not obvious on CECT (case 6). Some previous studies or cases suggest that MTWLS are mostly solid masses (1, 3, 6), which is inconsistent with our findings. However, as increasingly many cases have been reported, MTWLS has become more likely to have cystic components. Many of the latest pathological studies (Oramas et al. (7) [25 cases, 100%]; Hulme et al. (8) [4 cases, 80%]; Liu et al. (9) [4 cases, 100%]) believe that MTWLS has a high rate of cystic changes, which is consistent with our study. These latest studies provide a pathological basis for the high rate of cystic changes in MTWLS. The shift in these findings suggests that an increased rate of cystic change may be a new sign that is gradually being recognized at both pathological and imaging levels. We are the first study to reflect these features on CECT.

Previous studies have shown that with a higher risk of thymoma, it is more likely to contain cystic/necrotic components (10–12). However, MTWLS has no obvious malignant biological behavior but is more likely to have cystic components, which is noteworthy in the differential diagnosis. Additionally, attention should be given to the local invasiveness of the tumor to distinguish MTWLS from high-risk thymoma and thymic carcinoma. CECT can be used to identify the cystic components of the case, which is helpful for the diagnosis of this disease.

In this study, seven patients showed significant enhancement, among whom four patients had a consistent imaging appearance with annular enhancement, since the cystic component was located in the center. Pathologically, one explanation is that the solid component of the tumor is the medullary thymic epithelium with abundant lymphoid stroma and surrounding blood supply, which makes the enhancement more significant (13). Previous studies have found that the maximal contrast-enhanced range (CE_max_) of low-risk thymoma (A, AB) is significantly higher than that of high-risk thymoma (B1, B2, B3, thymic carcinoma) (10, 14). The relatively benign biological behavior of MTWLS is consistent with previous studies. Other tumors of nonthymic origin may also appear in the anterior mediastinum. Similar to MTWLS, they may have a cystic component with a markedly enhanced solid component, such as cystic teratoma and substernal goiter. We can make differential diagnoses from clinical data and other imaging features. Cystic teratoma may have fat and bone components inside; substernal goiter can be determined by whether it is connected to the thyroid or very close to the enhancement pattern of the thyroid (15).

This study shows that MTWLS is more common in middle-aged and elderly people (average age 61.4 years, range 54-72 years) and is mostly found by physical examination and usually without obvious clinical symptoms, such as myasthenia gravis (0/10; 0%). The surgical prognosis of the disease was good, and no recurrence was found in any patients in the study (average follow-up time was 3.9 years), which is consistent with the known relatively benign biological behavior of MTWLS.

This study has certain limitations. First, this study is a retrospective study, and the CT scan parameters of the enrolled cases were slightly different, which may have affected the measurements of the CT value. Second, the number of cases enrolled in this study was relatively small. Since the incidence of MTWLS itself is very low, further research with larger samples and multi-center data is needed.

In summary, this study presents the CECT imaging features of MTWLS for the first time. MTWLS is mostly cystic-solid, with multiple cystic components, the solid components are significantly enhanced, and there are no signs of invasion or other malignant signs on CECT. We hope to draw more convincing conclusions with the support of more cases in the future.

## Data availability statement

The original contributions presented in the study are included in the article/**Supplementary Material**. Further inquiries can be directed to the corresponding author.

## Ethics statement

The studies involving human participants were reviewed and approved by Cancer Hospital, Chinese Academy of Medical Sciences. Written informed consent for participation was not required for this study in accordance with the national legislation and the institutional requirements.

## Author contributions

Guarantors of integrity of entire study: ML; study concept/design: ML; data acquisition or data analysis/interpretation: all authors; manuscript drafting or revision for important intellectual content, all authors; appropriate resolution of any questions related to the work: all authors; literature research: LM and L-LQ.; clinical studies: LM, X-JS, and J-XZ; statistical analysis: LM and H-HZ; and manuscript editing: LM, ML, and LY. All authors contributed to the article and approved the submitted version.

## Acknowledgments

We appreciate the investigators at all participating study sites.

## Conflict of interest

The authors declare that the research was conducted in the absence of any commercial or financial relationships that could be construed as a potential conflict of interest.

## Publisher’s note

All claims expressed in this article are solely those of the authors and do not necessarily represent those of their affiliated organizations, or those of the publisher, the editors and the reviewers. Any product that may be evaluated in this article, or claim that may be made by its manufacturer, is not guaranteed or endorsed by the publisher.

## Abbreviations

**MTWLS,** Micronodular thymoma with lymphoid stroma; **CECT**, Contrast-enhanced computed tomography; **MNTCLH**, Micronodular thymic carcinoma with lymphoid hyperplasia; **VATS**, Video-assisted thoracic surgery; **CE_max_**, Maximal contrast-enhanced range; **AJCC**, American Joint Committee on Cancer.
