# Supplementary material for: Micronodular thymoma with lymphoid stroma: Contrast-enhanced CT features with histopathological correlation in 10 patients
Source: Front Oncol. 2022 Aug 30;12:964882. doi: 10.3389/fonc.2022.964882 (PMC9468748; doi:10.3389/fonc.2022.964882)

**Supplementary materials include:**

**Table1: Supplementary Clinical Features of MTWLS**

**Table2: Supplementary Pathological Features of MTWLS**

**Table3: Supplementary Immunohistochemistry Features of MTWLS**

**Figure1~6: Supplementary Case (2, 5, 7, 8, 9, 10) images of MTWLS**

**Table1: Supplementary Clinical Features of MTWLS**

| Case | Discovery process | Hypertension | Smoking | Drinking | Family history | CA199 | AFP | CEA | β-HCG |
| --- | --- | --- | --- | --- | --- | --- | --- | --- | --- |
| 1 | Incidental finding on chest CT | - | - | - | Gastric cancer (father) | NA | - | - | - |
| 2 | Incidental finding on chest CT | - | ＋ | ＋ | - | NA | NA | NA | NA |
| 3 | Incidental finding on chest CT | ＋ | ＋ | ＋ | - | - | - | - | - |
| 4 | Lung cancer examination by CT | ＋ | ＋ | ＋ | - | NA | NA | - | NA |
| 5 | Incidental finding on chest CT | - | - | - | Lung cancer(grandmother) | NA | - | - | - |
| 6 | Incidental finding on chest CT | ＋ | ＋ | ＋ | - | NA | NA | NA | NA |
| 7 | Incidental finding on chest CT | - | ＋ | ＋ | Breast cancer (sister) | NA | NA | - | NA |
| 8 | Incidental finding on chest CT | ＋ | - | - | - | NA | - | NA | - |
| 9 | Incidental finding on chest CT | ＋ | - | - | NA | NA | NA | - | NA |
| 10 | Incidental finding on chest CT | ＋ | - | - | - | NA | NA | - | NA |

**Table2: Supplementary Pathological Features of MTWLS**

| Case | Complete capsule | Capsular invasion | Cystic component | Surrounding fat invasion | Mediastinal pleural invasion | Masaoka |
| --- | --- | --- | --- | --- | --- | --- |
| 1 | NA | - | + | NA | - | 1 |
| 2 | NA | NA | + | - | - | 1 |
| 3 | + | NA | + | NA | - | 1 |
| 4 | NA | + | + | + | NA | 2 |
| 5 | NA | + | - | - | - | 2 |
| 6 | NA | + | + | + | NA | 2 |
| 7 | + | NA | + | - | NA | 1 |
| 8 | + | NA | + | + | NA | 2 |
| 9 | NA | + | NA | + | NA | 2 |
| 10 | NA | NA | + | NA | NA | 1 |

**Table3: Supplementary Immunohistochemistry Features of MTWLS**

| Case | CD117 | CK19 | CK7 | EMA | Ki-67 | P63 | CD5 | CD1a | TdT | CD3 | CD5 | CD20 |
| --- | --- | --- | --- | --- | --- | --- | --- | --- | --- | --- | --- | --- |
| 1 | NA | NA | NA | NA | NA | NA | NA | NA | NA | NA | NA | NA |
| 2 | - | 2+ | 3+ | - | 0.1 | 3+ | NA | + | + | + | + | NA |
| 3 | - | 1+ | NA | NA | 0.02 | 1+ | NA | + | + | + | + | + |
| 4 | - | 3＋ | 3＋ | NA | NA | 3+ | NA | + | + | + | + | NA |
| 5 | - | 3＋ | NA | - | + | 3+ | NA | + | + | + | + | + |
| 6 | - | 3＋ | 3+ | + | 0.15 | 3+ | NA | + | + | + | + | + |
| 7 | - | 3+ | 2+ | NA | 0.4 | 3+ | - | + | + | + | + | NA |
| 8 | NA | NA | NA | NA | NA | NA | NA | NA | NA | NA | NA | NA |
| 9 | NA | 3+ | 3+ | NA | 0.15 | NA | NA | + | + | NA | + | + |
| 10 | NA | NA | NA | NA | NA | NA | NA | NA | NA | NA | NA | NA |

**Figure1: Supplementary Case2 image of MTWLS**


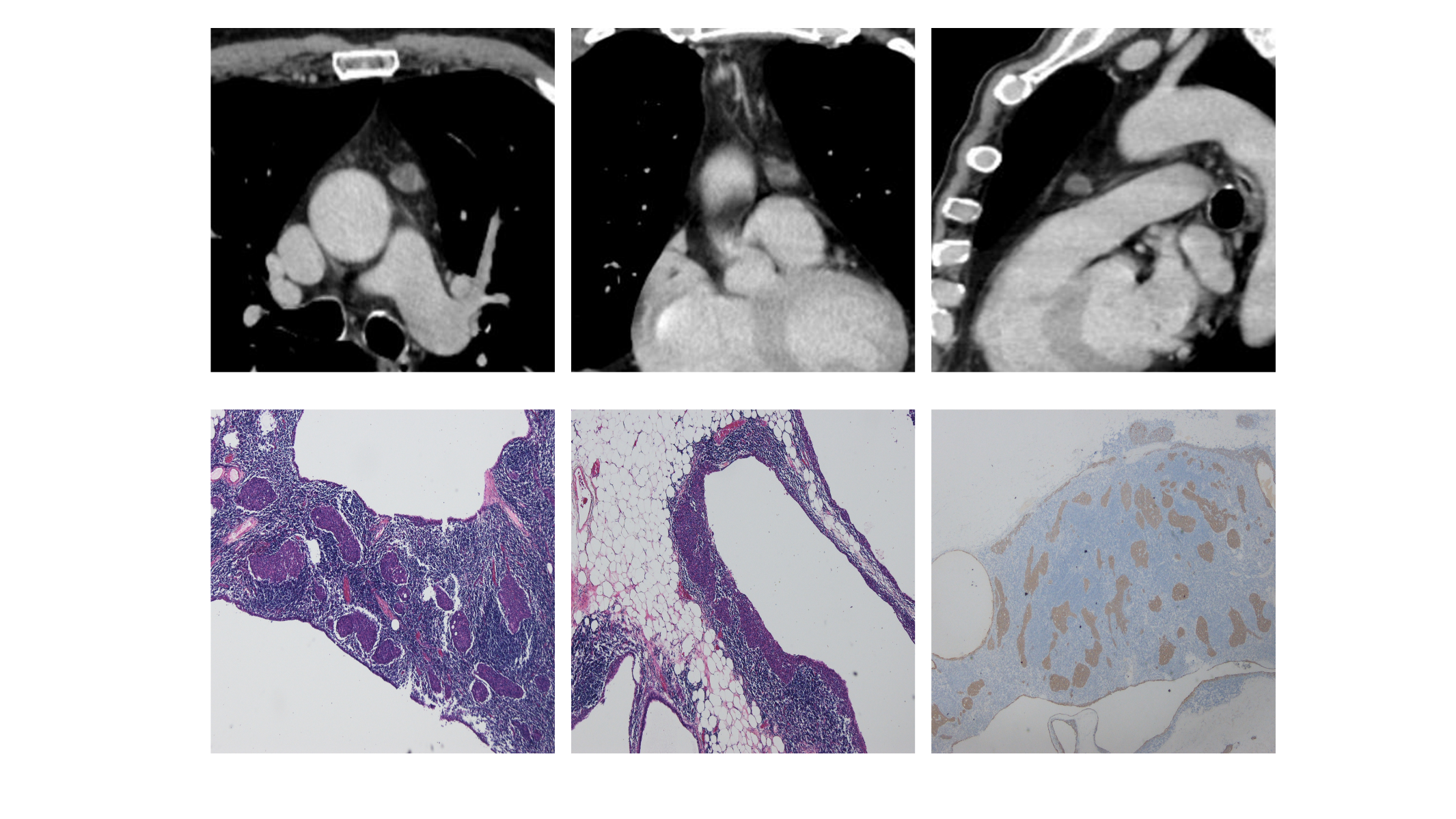


**Figure2: Supplementary Case5 image of MTWLS**


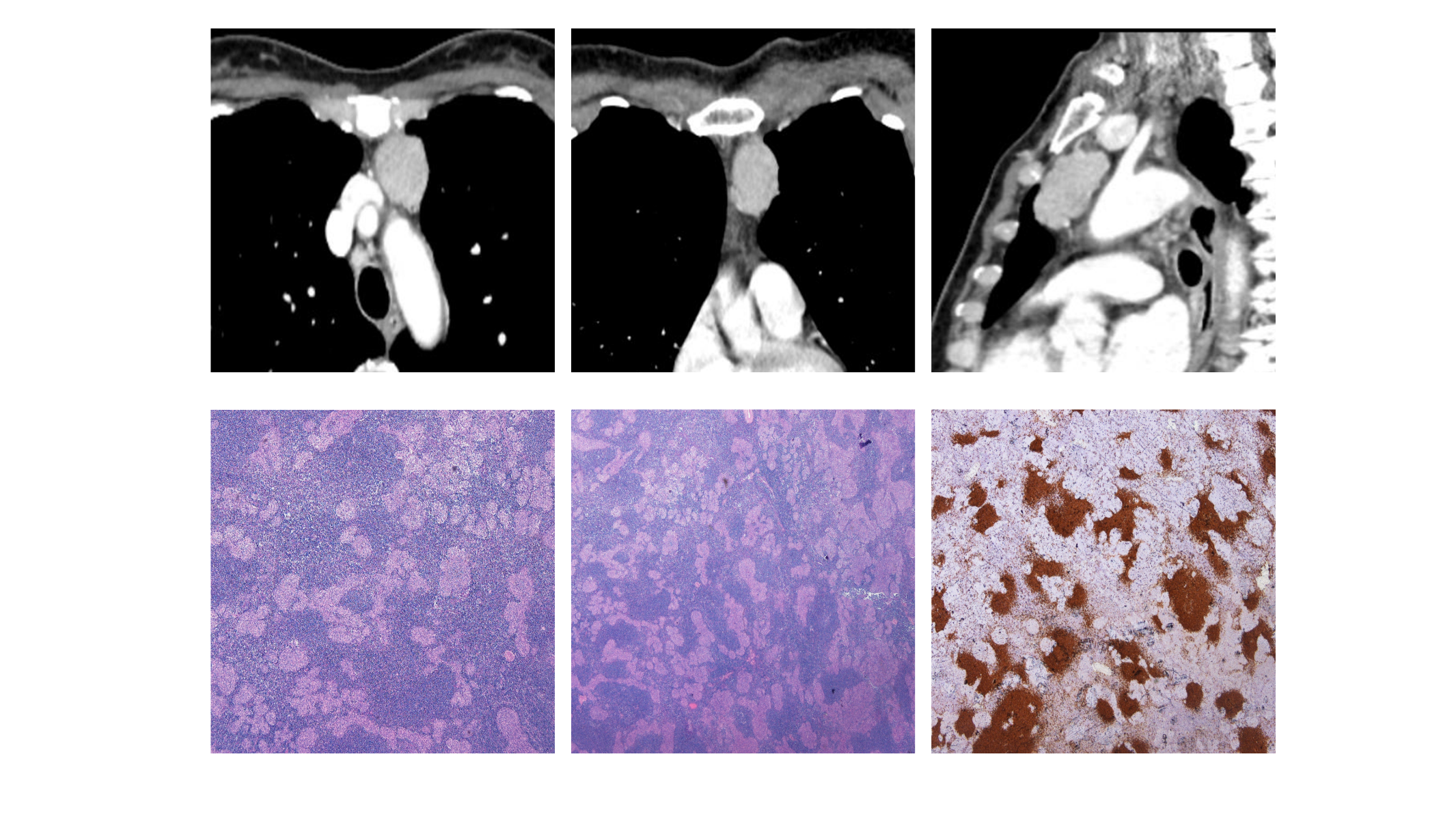


**Figure3: Supplementary Case7 image of MTWLS**


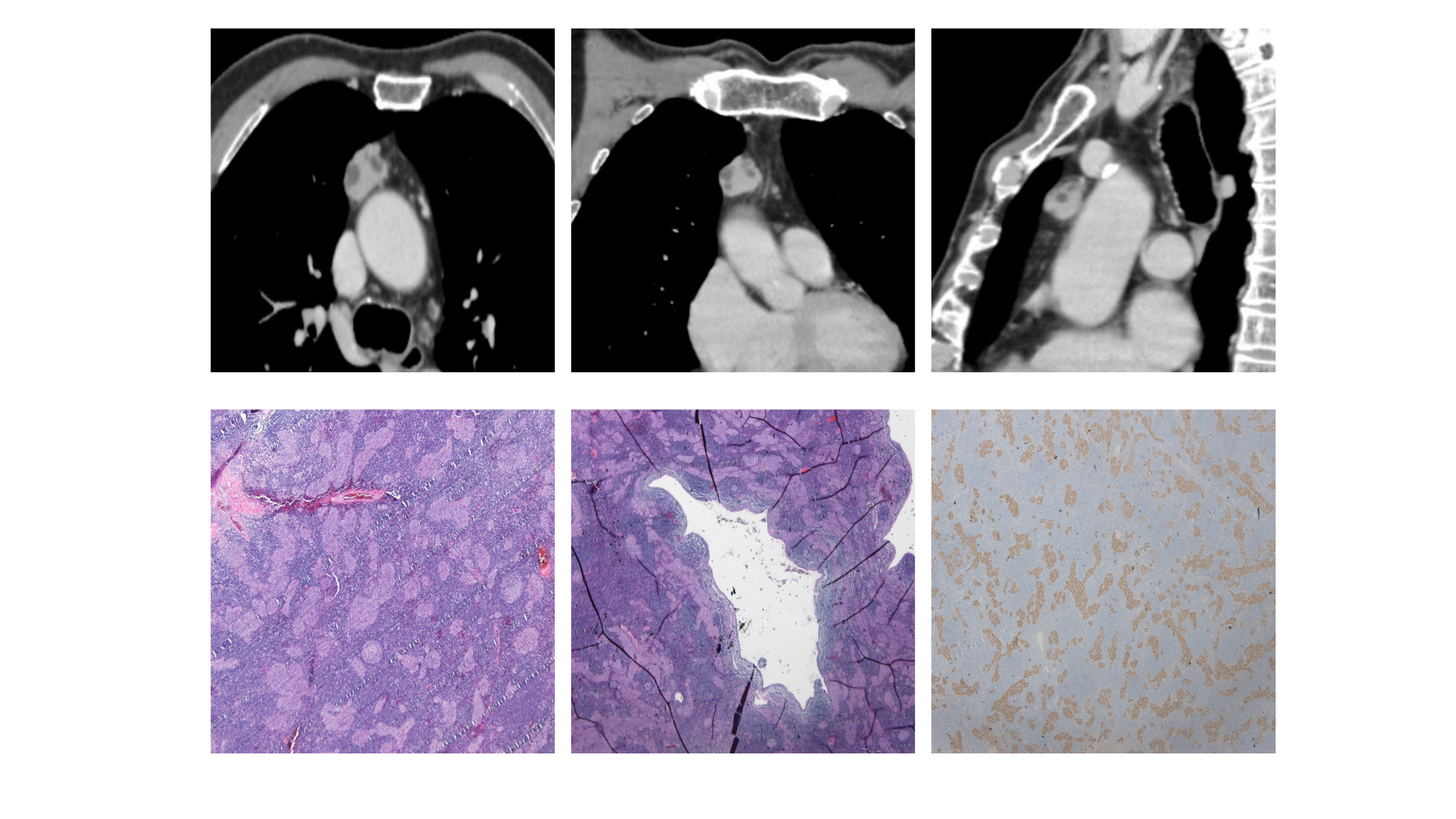


**Figure4: Supplementary Case8 image of MTWLS**


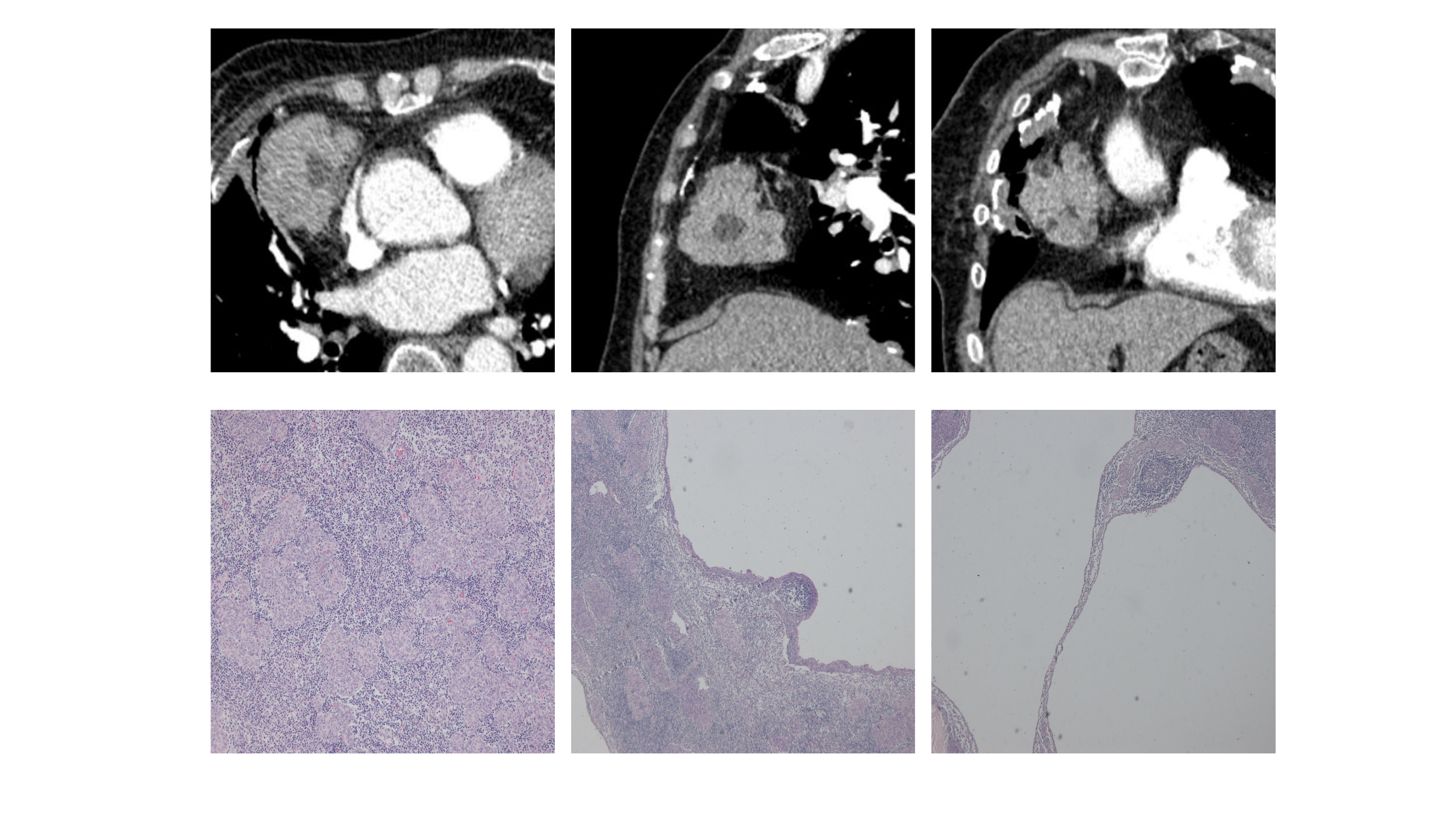


**Figure5: Supplementary Case9 image of MTWLS**


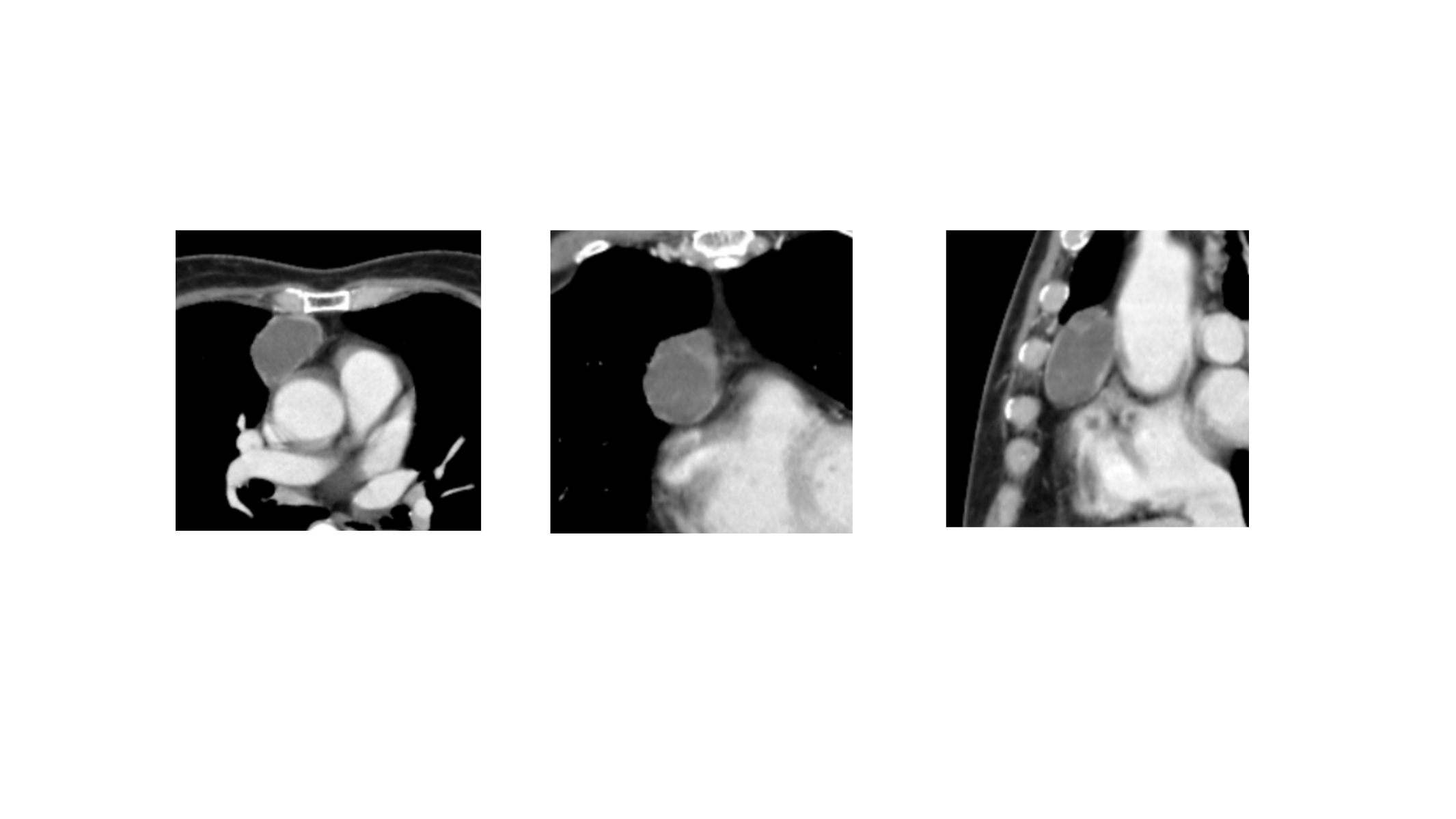


**Figure6: Supplementary Case10 image of MTWLS**


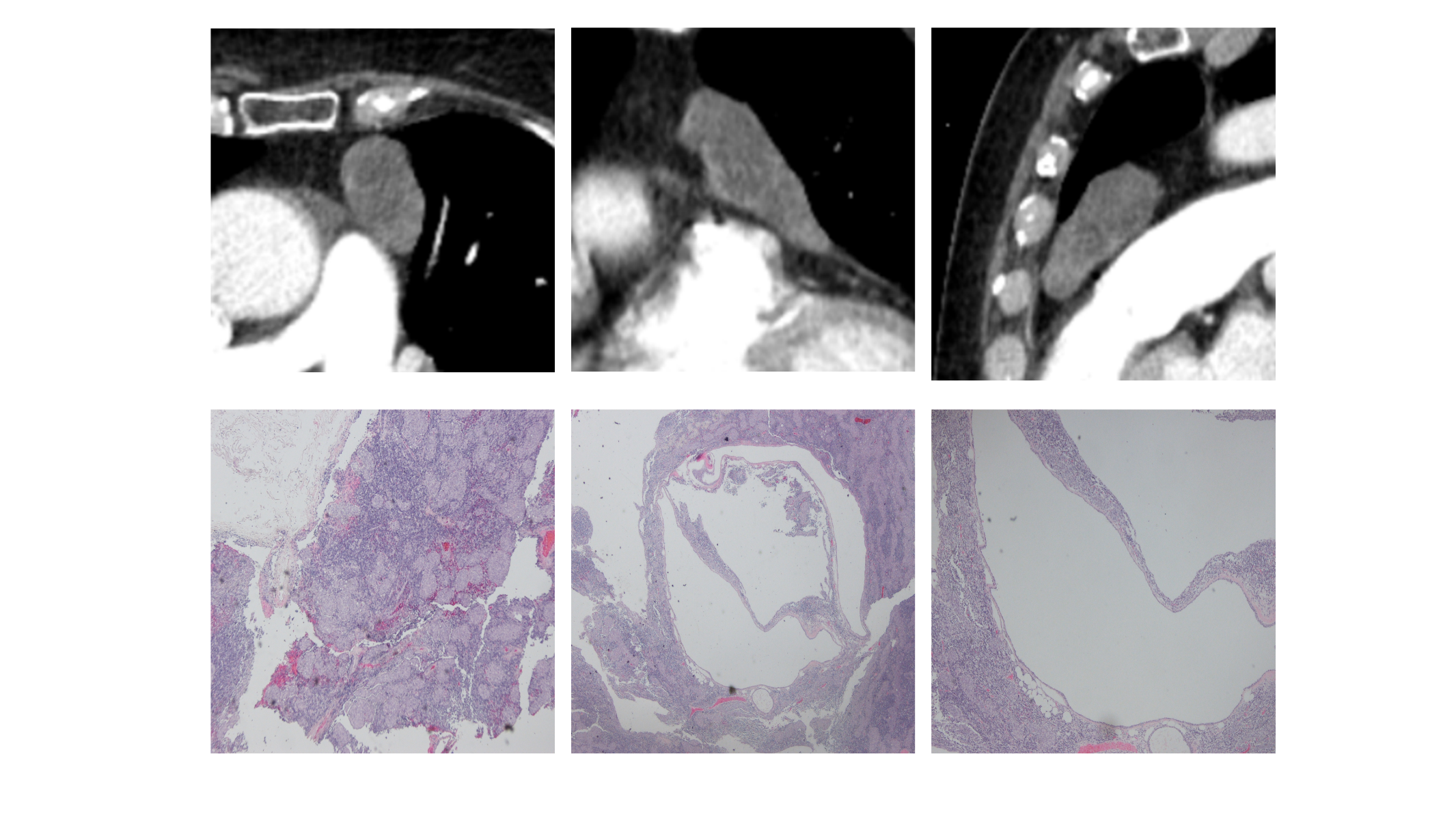

Supplement: Supplementary file 1 [file DataSheet_1.docx]
